# Crystal structure and Hirshfeld surface analysis of 4-{[(anthracen-9-yl)meth­yl]amino}­benzoic acid

**DOI:** 10.1107/S2056989019016207

**Published:** 2020-01-01

**Authors:** Adeeba Ahmed, Md. Serajul Haque Faizi, Aiman Ahmad, Musheer Ahmad, Igor O. Fritsky

**Affiliations:** a Department of Applied Chemistry, ZHCET, Aligarh Muslim University, Aligarh 202002 (UP), India; b Department of Chemistry, Langat Singh College, B. R. A. Bihar University, Muzaffarpur, Bihar 842001, India; c Taras Shevchenko National University of Kyiv, Department of Chemistry, 64 Vladimirska Str., Kiev 01601, Ukraine

**Keywords:** crystal structure, 4-amino­benzoic acid (PABA), 9-anthraldehyde, hydrogen bonding, C—H⋯π inter­actions

## Abstract

In the mol­ecule of the title anthracene derivative, C_22_H_17_NO_2_, the benzene ring is inclined to the mean plane of the anthracene ring system (r.m.s. deviation = 0.024 Å) by 75.21 (9)°. In the crystal, mol­ecules are linked by pairs of O—H⋯O hydrogen bonds, forming classical carb­oxy­lic acid inversion dimers with an *R* ^2^_2_(8) ring motif. The dimers are linked by C—H⋯π inter­actions, forming a supra­molecular framework.

## Abstract 2

In mol­ecule of the title compound, the benzene ring is inclined to the mean plane of the anthracene ring system (r.m.s. deviation = 0.024 Å) by 75.21 (9)°. In the crystal, a classical carb­oxy­lic acid inversion dimer is formed enclosing an 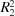
(8) ring motif.

## Chemical context   

Anthraldehyde has been used in the synthesis of several Schiff base compounds that exhibit fluorescent properties as a result of strong π–π conjugation (Asiri *et al.*, 2011 ▸; Pavitha *et al.*, 2017 ▸). Many complexes synthesized using anthraldehyde have shown remarkable sensing properties and have been used as chemo sensors (Obali & Ucan, 2012 ▸; Zhou *et al.*, 2012 ▸). Schiff base compounds are also of inter­est because of their biological applications, which include anti­bacterial, anti­cancer and anti­viral (Asiri & Khan, 2010 ▸; Cheng *et al.*, 2010 ▸) activities. Herein, we report on the crystal and mol­ecular structures of the title Schiff base compound, 4-{[(anthracen-9-yl)meth­yl]amino}­benzoic acid, synthesized *via* reaction of 9-anthraldehyde with 4-amino­benzoic acid (PABA) followed by reduction with sodium borohydride.
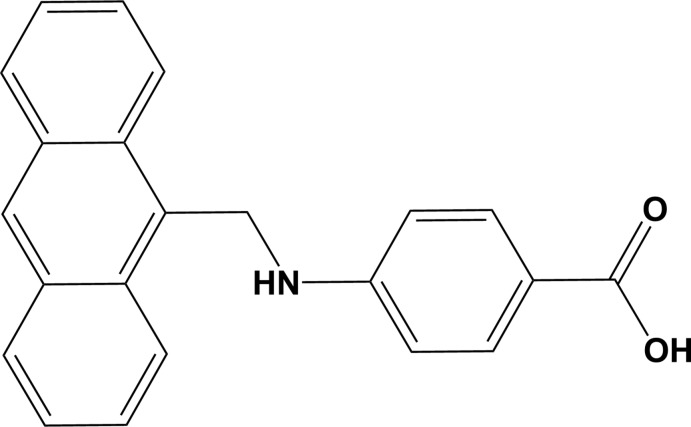


## Structural commentary   

**Figure 1:**
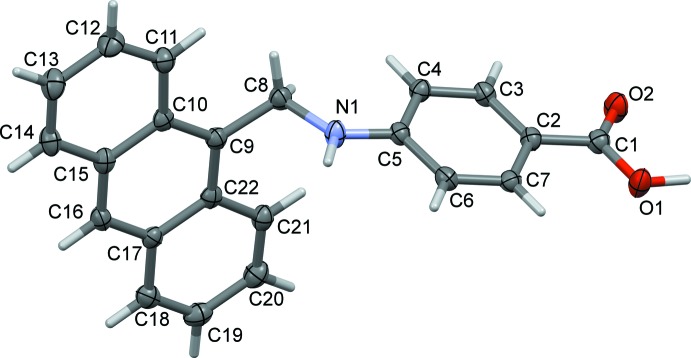
The mol­ecular structure of the tittle compound, with atom labelling. Displacement ellipsoids are drawn at the 50% probability level.

The mol­ecular structure of the title compound is illustrated in Fig. 1 ▸. The mol­ecule is non-planar, with the benzene ring (C2–C7) being inclined to the mean plane of the anthracene ring system (C9–C22; r.m.s. deviation = 0.024 Å) by 75.21 (9)°, and the torsional angle of the bridge, C5—N1—C8—C9, is 142.6 (2)°. The C8—N1 bond length of 1.457 (3) Å, is comparable to the C—N bond-length values obtained for the similar ligand 5-[(anthracen-9-ylmeth­yl)amino]­isophthalic acid (see §5. *Database survey*).

The C1=O2 and C1—O1 bond lengths of 1.238 (3) and 1.325 (3) Å, respectively, are in the expected ranges (Cambridge Structural Database; Groom *et al.*, 2016 ▸).

## Supra­molecular features   

**Figure 2:**
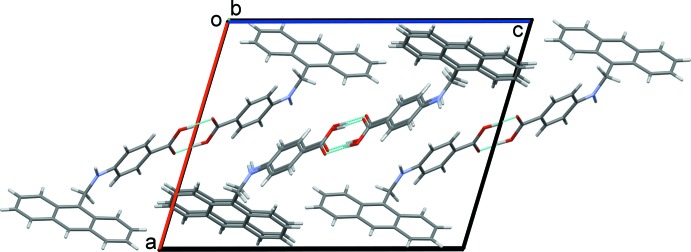
A partial view along the *b* axis of crystal packing of the title compound. The hydrogen bonds (Table 1 ▸) are shown as dashed lines.

**Figure 3:**
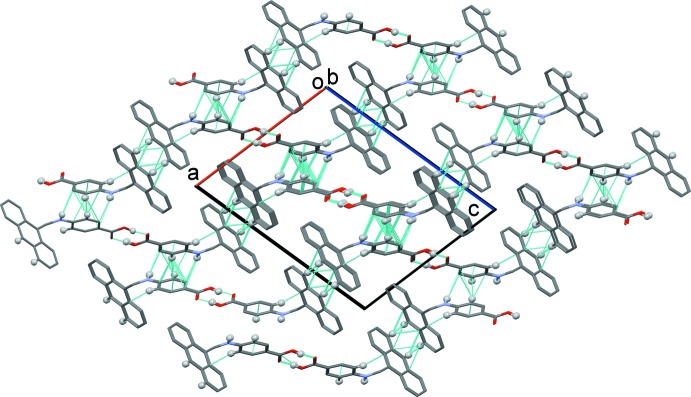
A view along the *b* axis of crystal packing of the title compound. The O—H⋯O hydrogen bonds and the C—H⋯π inter­actions are indicated by dashed lines (Table 1 ▸). For clarity, only the H atoms (grey balls) involved in these inter­actions have been included.

In the crystal, a classical carb­oxy­lic acid inversion dimer is formed enclosing an 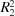
(8) ring motif (Table 1 ▸ and Fig. 2 ▸). The dimers pack along the *a*-axis direction in a herringbone fashion. They are linked by a series of C—H⋯π inter­actions (Table 1 ▸ and Fig. 3 ▸), forming a supra­molecular three-dimensional structure. The NH hydrogen atom (H1*A*) is not involved in hydrogen bonding but is directed towards the benzene ring (C2–C7). Approximate geometrical details of this weak N—H⋯π inter­action are given in Table 1 ▸.

## Hirshfeld analysis   

The Hirshfeld surface analysis (Spackman & Jayatilaka, 2009 ▸) and the associated two-dimensional fingerprint plots (McKinnon *et al.*, 2007 ▸) were performed with *CrystalExplorer17* (Turner *et al.*, 2017 ▸). The Hirshfeld surfaces are colour-mapped with the normalized contact distance, *d*_norm_, from red (distances shorter than the sum of the van der Waals radii) through white to blue (distances longer than the sum of the van der Waals radii).

**Figure 4:**
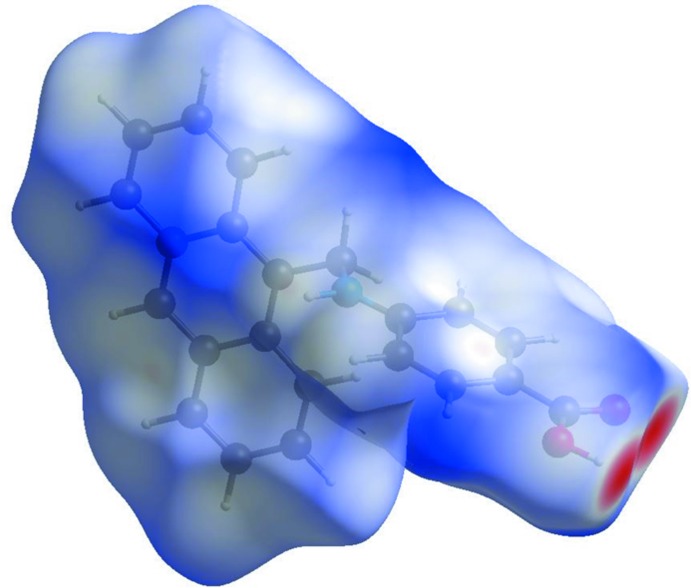
The Hirshfeld surface of the title compound mapped over *d*_norm_, in the colour range −0.7519 to 1.6997 a.u..

The Hirshfeld surface of the title compound mapped over *d*_norm_, in the colour range −0.7519 to 1.6997 a.u., is given in Fig. 4 ▸. The positions of the strong O—H⋯O hydrogen bonds are indicated by the red regions on the Hirshfeld surface.

**Figure 5:**
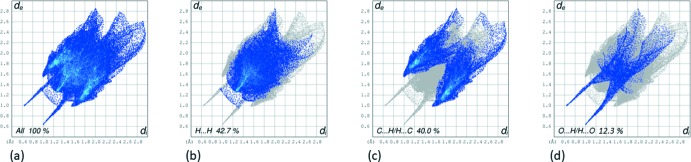
(*a*) The two-dimensional fingerprint plots of the title compound, and delineated into (*b*) H⋯H (42.7%), (*c*) C⋯H/H⋯C (40.0%) and (*d*) O⋯H/H⋯O (12.3%) contacts.

The two-dimensional fingerprint plots are given in Fig. 5 ▸. They reveal that the principal contributions to the overall surface involve H⋯H contacts at 42.7% (Fig. 5 ▸ *b*), followed by C⋯H/H⋯C contacts at 40.0% (Fig. 5 ▸ *c*) and O⋯H/H⋯O contacts at 12.3% (Fig. 5 ▸ *d*). Apart from the C⋯C contacts, contributing 2.1%, all other atom⋯atom contact contributions are negligible.

## Database survey   

A search of the Cambridge Structural Database (CSD, Version 5.40, update August 2019; Groom *et al.*, 2016 ▸) for the *N*-(anthracen-9-ylmeth­yl)aniline skeleton gave six hits (see supporting information file S1), all of which concern polymeric metal complexes of the ligand 5-[(anthracen-9-ylmeth­yl)amino]­isophthalic acid; for example, a series of four gadolinium coordination polymers (CSD refcodes VOLSOG, VOLSUM, VOLTAT, VOLTIB; Singh *et al.*, 2014 ▸). The bridging C—N bond length varies from *ca*.1.389 to 1.494 Å, compared to the C8—N1 bond length of 1.457 (3) Å in the title compound.

A search for the 1-(anthracen-9-yl)-*N*-phenyl­methanimine skeleton gave 21 hits (see supporting information file S2), none of which involve a benzoic acid moiety.

## Synthesis and crystallization   

4-Amino­benzoic acid (0.33 g, 2.42 mmol) was added to a solution of 9-anthraldehyde (0.5 g, 2.42 mmol) dissolved in ethanol and the whole mixture was heated at 343 K under reflux for 5–6 h. The mixture was then stirred for a further 10 h at room temperature to obtain a yellow precipitate of the new product, which was monitored through TLC. The yellow precipitate, which was then air dried, was obtained in 76% yield. This was further reduced with sodium borohydride taken in excess (0.183 g, 4.84 mmol) by maintaining the temperature at 277–278 K until the colour of the precipitate had changed from bright yellow to dull yellow. The precipitate was filtered, washed with water and acidified with acetic acid. The product thus obtained was dissolved in hot ethanol and kept for crystallization. Block-like pale-yellow crystals of the title compound were obtained after a few days.

## Refinement   

Crystal data, data collection and structure refinement details are summarized in Table 2 ▸. The OH and NH hydrogen atoms were located in a difference-Fourier map and refined freely. The C-bound H atoms were included in calculated positions and allowed to ride on their parent C atom: C—H = 0.93–0.97Å with *U*_iso_(H) = 1.2*U*_eq_(C).

## Supplementary Material

Crystal structure: contains datablock(s) Global, I. DOI: 10.1107/S2056989019016207/su5532sup1.cif

Structure factors: contains datablock(s) I. DOI: 10.1107/S2056989019016207/su5532Isup2.hkl

Supporting information file. DOI: 10.1107/S2056989019016207/su5532Isup5.cml

CSD search S1. DOI: 10.1107/S2056989019016207/su5532sup3.pdf

CSD search S2. DOI: 10.1107/S2056989019016207/su5532sup4.pdf

CCDC reference: 1969448

Additional supporting information:  crystallographic information; 3D view; checkCIF report

## Figures and Tables

**Table 1:** Hydrogen-bond geometry (Å, °) *Cg*1, *Cg*2, and *Cg*4 are the centroids of the C2–C7, C9/C10/C15–C17/C22 and C17–C22 rings, respectively. Approximative geometrical parameters are given for the weak N—H..π inter­action.

*D*—H⋯*A*	*D*—H	H⋯*A*	*D*⋯*A*	*D*—H⋯*A*
O1—H1⋯O2^i^	1.05 (4)	1.58 (3)	2.621 (3)	172 (3)
N1—H1A⋯*Cg*1^ii^	0.93 (3)	3.49	4.140	129
C4—H4⋯*Cg*4^iii^	0.93	2.98 (1)	3.752 (3)	141 (1)
C6—H6⋯*Cg*1^ii^	0.93	2.69 (1)	3.410 (3)	135 (1)
C16—H16⋯*Cg*4^iv^	0.93	2.83 (1)	3.644 (3)	147 (1)
C18—H18⋯*Cg*2^iv^	0.93	2.69 (1)	3.452 (3)	140 (1)

Symmetry codes: (i) 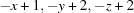; (ii) 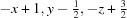; (iii) 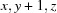; (iv) 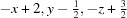.

**Table 2:** Experimental details

Crystal data	
Chemical formula	C_22_H_17_NO_2_
*M*_r_	327.39
Crystal system, space group	Monoclinic, *P*2_1_/*c*
Temperature (K)	100
*a*, *b*, *c* (Å)	14.985 (2), 6.0116 (9), 19.106 (3)
β (°)	106.796 (5)
*V* (Å^3^)	1647.7 (4)
*Z*	4
Radiation type	Mo *K*α
μ (mm^−1^)	0.09
Crystal size (mm)	0.4 × 0.27 × 0.18

Data collection	
Diffractometer	Bruker APEXII CCD
Absorption correction	Multi-scan (*SADABS*; Krause *et al.*, 2015 ▸)
*T*_min_, *T*_max_	0.629, 0.746
No. of measured, independent and observed [*I* > 2σ(*I*)] reflections	25595, 2913, 1975
*R*_int_	0.118
(sin θ/λ)_max_ (Å^−1^)	0.596

Refinement	
*R*[*F* ^2^ > 2σ(*F* ^2^)], *wR*(*F* ^2^), *S*	0.048, 0.141, 1.12
No. of reflections	2913
No. of parameters	235
H-atom treatment	H atoms treated by a mixture of independent and constrained refinement
Δρ_max_, Δρ_min_ (e Å^−3^)	0.38, −0.32

Computer programs: *APEX2* and *SAINT* (Bruker, 2016 ▸), *olex2.solve* (Bourhis *et al.*, 2015 ▸), *olex2.refine* (Bourhis *et al.*, 2015 ▸), *OLEX2* (Dolomanov *et al.*, 2009 ▸), *Mercury* (Macrae *et al.*, 2008 ▸), *PLATON* (Spek, 2009 ▸) and *publCIF* (Westrip, 2010 ▸).

## supplementary crystallographic information

### Crystal data

**Table:**

C_22_H_17_NO_2_	*F*(000) = 688.3239
*M**_r_* = 327.39	*D*_x_ = 1.320 Mg m^−^^3^
Monoclinic, *P*2_1_/*c*	Mo *K*α radiation, λ = 0.71073 Å
*a* = 14.985 (2) Å	Cell parameters from 3139 reflections
*b* = 6.0116 (9) Å	θ = 3.1–28.2°
*c* = 19.106 (3) Å	µ = 0.09 mm^−^^1^
β = 106.796 (5)°	*T* = 100 K
*V* = 1647.7 (4) Å^3^	Block, pale-yellow
*Z* = 4	0.4 × 0.27 × 0.18 mm

### Data collection

**Table:**

Bruker APEXII CCD diffractometer	1975 reflections with *I* > 2σ(*I*)
φ and ω scans	*R*_int_ = 0.118
Absorption correction: multi-scan (SADABS; Krause *et al.*, 2015)	θ_max_ = 25.1°, θ_min_ = 2.8°
*T*_min_ = 0.629, *T*_max_ = 0.746	*h* = −20→20
25595 measured reflections	*k* = −8→8
2913 independent reflections	*l* = −25→25

### Refinement

**Table:**

Refinement on *F*^2^	29 constraints
Least-squares matrix: full	Primary atom site location: iterative
*R*[*F*^2^ > 2σ(*F*^2^)] = 0.048	H atoms treated by a mixture of independent and constrained refinement
*wR*(*F*^2^) = 0.141	*w* = 1/[σ^2^(*F*_o_^2^) + (0.0452*P*)^2^ + 0.9105*P*] where *P* = (*F*_o_^2^ + 2*F*_c_^2^)/3
*S* = 1.12	(Δ/σ)_max_ = 0.0003
2913 reflections	Δρ_max_ = 0.38 e Å^−^^3^
235 parameters	Δρ_min_ = −0.32 e Å^−^^3^
0 restraints	

### Fractional atomic coordinates and isotropic or equivalent isotropic displacement parameters (Å^2^)

**Table:**

	*x*	*y*	*z*	*U*_iso_*/*U*_eq_	
O1	0.47155 (12)	0.7404 (3)	0.95012 (10)	0.0304 (5)	
O2	0.57543 (12)	1.0125 (3)	0.95315 (10)	0.0317 (5)	
N1	0.66609 (14)	0.3342 (4)	0.72544 (12)	0.0252 (5)	
C1	0.54000 (17)	0.8318 (4)	0.92904 (14)	0.0238 (6)	
C2	0.57055 (16)	0.7035 (4)	0.87497 (13)	0.0212 (6)	
C3	0.63813 (16)	0.7902 (4)	0.84570 (14)	0.0230 (6)	
H3	0.66266 (16)	0.9303 (4)	0.86054 (14)	0.0276 (7)*	
C4	0.66973 (17)	0.6741 (4)	0.79531 (13)	0.0222 (6)	
H4	0.71436 (17)	0.7367 (4)	0.77612 (13)	0.0266 (7)*	
C5	0.63421 (16)	0.4606 (4)	0.77302 (13)	0.0209 (6)	
C6	0.56515 (17)	0.3731 (4)	0.80193 (13)	0.0224 (6)	
H6	0.53997 (17)	0.2335 (4)	0.78701 (13)	0.0269 (7)*	
C7	0.53463 (16)	0.4920 (4)	0.85202 (13)	0.0215 (6)	
H7	0.48944 (16)	0.4312 (4)	0.87096 (13)	0.0258 (7)*	
C8	0.74688 (17)	0.3903 (4)	0.70105 (14)	0.0243 (6)	
H8a	0.72642 (17)	0.4653 (4)	0.65418 (14)	0.0291 (7)*	
H8b	0.78696 (17)	0.4913 (4)	0.73586 (14)	0.0291 (7)*	
C9	0.80133 (16)	0.1835 (4)	0.69386 (13)	0.0208 (6)	
C10	0.80253 (16)	0.1014 (4)	0.62496 (13)	0.0204 (6)	
C11	0.75646 (17)	0.2083 (4)	0.55691 (14)	0.0257 (6)	
H11	0.72308 (17)	0.3384 (4)	0.55740 (14)	0.0308 (7)*	
C12	0.76034 (18)	0.1242 (5)	0.49175 (14)	0.0294 (7)	
H12	0.72945 (18)	0.1972 (5)	0.44864 (14)	0.0353 (8)*	
C13	0.81084 (18)	−0.0732 (5)	0.48869 (15)	0.0310 (7)	
H13	0.81360 (18)	−0.1278 (5)	0.44382 (15)	0.0372 (8)*	
C14	0.85503 (17)	−0.1822 (4)	0.55102 (14)	0.0269 (6)	
H14	0.88740 (17)	−0.3124 (4)	0.54841 (14)	0.0322 (7)*	
C15	0.85289 (16)	−0.1007 (4)	0.62098 (14)	0.0216 (6)	
C16	0.89943 (16)	−0.2114 (4)	0.68500 (14)	0.0225 (6)	
H16	0.93082 (16)	−0.3429 (4)	0.68203 (14)	0.0270 (7)*	
C17	0.90025 (16)	−0.1302 (4)	0.75358 (13)	0.0202 (6)	
C18	0.94936 (16)	−0.2430 (4)	0.81929 (14)	0.0255 (6)	
H18	0.98095 (16)	−0.3741 (4)	0.81619 (14)	0.0307 (7)*	
C19	0.95099 (18)	−0.1630 (4)	0.88627 (15)	0.0289 (7)	
H19	0.98374 (18)	−0.2383 (4)	0.92840 (15)	0.0347 (8)*	
C20	0.90250 (17)	0.0354 (4)	0.89133 (15)	0.0287 (6)	
H20	0.90346 (17)	0.0897 (4)	0.93712 (15)	0.0344 (8)*	
C21	0.85437 (17)	0.1482 (4)	0.83019 (14)	0.0243 (6)	
H21	0.82321 (17)	0.2784 (4)	0.83510 (14)	0.0291 (7)*	
C22	0.85075 (16)	0.0706 (4)	0.75862 (14)	0.0204 (6)	
H1	0.457 (2)	0.850 (6)	0.9884 (19)	0.074 (11)*	
H1a	0.635 (2)	0.201 (5)	0.7113 (15)	0.042 (9)*	

### Atomic displacement parameters (Å^2^)

**Table:**

	*U*^11^	*U*^22^	*U*^33^	*U*^12^	*U*^13^	*U*^23^
O1	0.0321 (10)	0.0298 (10)	0.0357 (12)	−0.0061 (8)	0.0198 (9)	−0.0083 (9)
O2	0.0368 (11)	0.0274 (10)	0.0352 (12)	−0.0070 (9)	0.0172 (9)	−0.0115 (9)
N1	0.0215 (12)	0.0251 (12)	0.0322 (14)	−0.0041 (10)	0.0127 (10)	−0.0071 (10)
C1	0.0234 (14)	0.0238 (14)	0.0239 (15)	0.0007 (11)	0.0065 (11)	0.0001 (11)
C2	0.0200 (13)	0.0223 (13)	0.0209 (14)	0.0021 (11)	0.0053 (11)	−0.0011 (11)
C3	0.0204 (13)	0.0206 (13)	0.0275 (15)	0.0013 (11)	0.0059 (11)	−0.0024 (11)
C4	0.0197 (13)	0.0236 (13)	0.0242 (14)	−0.0003 (11)	0.0078 (11)	0.0018 (11)
C5	0.0195 (13)	0.0207 (13)	0.0219 (14)	0.0026 (10)	0.0051 (11)	0.0004 (11)
C6	0.0215 (13)	0.0194 (13)	0.0251 (15)	0.0012 (11)	0.0050 (11)	−0.0012 (11)
C7	0.0185 (13)	0.0229 (13)	0.0230 (14)	0.0015 (11)	0.0058 (11)	0.0036 (11)
C8	0.0234 (13)	0.0218 (13)	0.0295 (16)	−0.0009 (11)	0.0105 (12)	−0.0011 (11)
C9	0.0193 (13)	0.0202 (13)	0.0241 (14)	−0.0013 (10)	0.0081 (11)	−0.0007 (11)
C10	0.0147 (12)	0.0227 (13)	0.0242 (15)	−0.0019 (10)	0.0063 (11)	−0.0005 (11)
C11	0.0216 (13)	0.0277 (14)	0.0279 (15)	0.0013 (11)	0.0076 (11)	0.0009 (12)
C12	0.0250 (14)	0.0397 (16)	0.0215 (15)	0.0021 (12)	0.0037 (12)	0.0020 (12)
C13	0.0267 (14)	0.0393 (16)	0.0258 (16)	0.0018 (13)	0.0057 (12)	−0.0067 (13)
C14	0.0238 (13)	0.0287 (14)	0.0276 (15)	0.0006 (12)	0.0066 (12)	−0.0089 (12)
C15	0.0170 (12)	0.0235 (13)	0.0239 (15)	−0.0030 (10)	0.0054 (11)	−0.0043 (11)
C16	0.0202 (13)	0.0190 (13)	0.0297 (15)	0.0000 (11)	0.0094 (11)	−0.0029 (11)
C17	0.0171 (12)	0.0212 (13)	0.0235 (14)	−0.0022 (10)	0.0076 (11)	0.0009 (11)
C18	0.0195 (13)	0.0249 (14)	0.0318 (16)	−0.0010 (11)	0.0066 (11)	0.0024 (12)
C19	0.0234 (14)	0.0345 (16)	0.0272 (16)	−0.0043 (12)	0.0047 (11)	0.0064 (12)
C20	0.0285 (15)	0.0338 (15)	0.0245 (16)	−0.0051 (12)	0.0087 (12)	−0.0032 (12)
C21	0.0216 (13)	0.0261 (14)	0.0268 (15)	−0.0023 (11)	0.0096 (11)	−0.0029 (12)
C22	0.0181 (12)	0.0203 (13)	0.0242 (14)	−0.0054 (10)	0.0085 (11)	−0.0030 (11)

### Geometric parameters (Å, º)

**Table:**

O1—C1	1.325 (3)	C10—C15	1.443 (3)
O1—H1	1.05 (4)	C11—H11	0.9300
O2—C1	1.238 (3)	C11—C12	1.360 (3)
N1—C5	1.372 (3)	C12—H12	0.9300
N1—C8	1.457 (3)	C12—C13	1.418 (4)
N1—H1a	0.92 (3)	C13—H13	0.9300
C1—C2	1.465 (3)	C13—C14	1.353 (4)
C2—C3	1.392 (3)	C14—H14	0.9300
C2—C7	1.401 (3)	C14—C15	1.433 (3)
C3—H3	0.9300	C15—C16	1.390 (3)
C3—C4	1.379 (3)	C16—H16	0.9300
C4—H4	0.9300	C16—C17	1.395 (3)
C4—C5	1.408 (3)	C17—C18	1.429 (3)
C5—C6	1.408 (3)	C17—C22	1.435 (3)
C6—H6	0.9300	C18—H18	0.9300
C6—C7	1.375 (3)	C18—C19	1.360 (4)
C7—H7	0.9300	C19—H19	0.9300
C8—H8a	0.9700	C19—C20	1.414 (4)
C8—H8b	0.9700	C20—H20	0.9300
C8—C9	1.515 (3)	C20—C21	1.363 (3)
C9—C10	1.411 (3)	C21—H21	0.9300
C9—C22	1.418 (3)	C21—C22	1.431 (3)
C10—C11	1.436 (3)		
			
H1—O1—C1	106.6 (18)	H11—C11—C10	119.13 (14)
C8—N1—C5	124.4 (2)	C12—C11—C10	121.7 (2)
H1a—N1—C5	115.7 (18)	C12—C11—H11	119.13 (16)
H1a—N1—C8	119.8 (18)	H12—C12—C11	119.57 (16)
O2—C1—O1	122.5 (2)	C13—C12—C11	120.9 (3)
C2—C1—O1	115.1 (2)	C13—C12—H12	119.57 (16)
C2—C1—O2	122.5 (2)	H13—C13—C12	119.99 (16)
C3—C2—C1	119.9 (2)	C14—C13—C12	120.0 (3)
C7—C2—C1	121.9 (2)	C14—C13—H13	119.99 (16)
C7—C2—C3	118.2 (2)	H14—C14—C13	119.39 (16)
H3—C3—C2	119.07 (15)	C15—C14—C13	121.2 (2)
C4—C3—C2	121.9 (2)	C15—C14—H14	119.39 (15)
C4—C3—H3	119.07 (15)	C14—C15—C10	119.3 (2)
H4—C4—C3	120.11 (15)	C16—C15—C10	119.6 (2)
C5—C4—C3	119.8 (2)	C16—C15—C14	121.1 (2)
C5—C4—H4	120.11 (14)	H16—C16—C15	119.16 (14)
C4—C5—N1	122.1 (2)	C17—C16—C15	121.7 (2)
C6—C5—N1	119.3 (2)	C17—C16—H16	119.16 (14)
C6—C5—C4	118.6 (2)	C18—C17—C16	121.5 (2)
H6—C6—C5	119.71 (14)	C22—C17—C16	119.5 (2)
C7—C6—C5	120.6 (2)	C22—C17—C18	119.0 (2)
C7—C6—H6	119.71 (15)	H18—C18—C17	119.22 (15)
C6—C7—C2	121.0 (2)	C19—C18—C17	121.6 (2)
H7—C7—C2	119.49 (14)	C19—C18—H18	119.22 (16)
H7—C7—C6	119.49 (15)	H19—C19—C18	120.26 (16)
H8a—C8—N1	109.44 (13)	C20—C19—C18	119.5 (3)
H8b—C8—N1	109.44 (13)	C20—C19—H19	120.26 (16)
H8b—C8—H8a	108.0	H20—C20—C19	119.47 (16)
C9—C8—N1	111.0 (2)	C21—C20—C19	121.1 (3)
C9—C8—H8a	109.44 (14)	C21—C20—H20	119.47 (16)
C9—C8—H8b	109.44 (13)	H21—C21—C20	119.29 (16)
C10—C9—C8	121.6 (2)	C22—C21—C20	121.4 (2)
C22—C9—C8	118.2 (2)	C22—C21—H21	119.29 (14)
C22—C9—C10	120.2 (2)	C17—C22—C9	119.6 (2)
C11—C10—C9	123.8 (2)	C21—C22—C9	123.0 (2)
C15—C10—C9	119.4 (2)	C21—C22—C17	117.4 (2)
C15—C10—C11	116.8 (2)		
			
C8—N1—C5—C4	8.8 (4)	C9—C10—C11—C12	179.3 (3)
C8—N1—C5—C6	−169.8 (2)	C15—C10—C11—C12	−0.3 (4)
C5—N1—C8—C9	142.6 (2)	C9—C10—C15—C14	−179.2 (2)
O1—C1—C2—C3	176.1 (2)	C9—C10—C15—C16	0.0 (4)
O1—C1—C2—C7	−4.8 (4)	C11—C10—C15—C14	0.4 (3)
O2—C1—C2—C3	−3.8 (4)	C11—C10—C15—C16	179.6 (2)
O2—C1—C2—C7	175.3 (2)	C10—C11—C12—C13	−0.3 (4)
C1—C2—C3—C4	179.2 (2)	C11—C12—C13—C14	0.8 (4)
C7—C2—C3—C4	0.1 (4)	C12—C13—C14—C15	−0.7 (4)
C1—C2—C7—C6	−179.1 (2)	C13—C14—C15—C10	0.1 (4)
C3—C2—C7—C6	0.0 (4)	C13—C14—C15—C16	−179.1 (3)
C2—C3—C4—C5	−0.8 (4)	C10—C15—C16—C17	−1.1 (4)
C3—C4—C5—N1	−177.2 (2)	C14—C15—C16—C17	178.0 (2)
C3—C4—C5—C6	1.4 (4)	C15—C16—C17—C18	−179.1 (2)
N1—C5—C6—C7	177.3 (2)	C15—C16—C17—C22	1.0 (4)
C4—C5—C6—C7	−1.4 (4)	C16—C17—C18—C19	179.4 (2)
C5—C6—C7—C2	0.7 (4)	C22—C17—C18—C19	−0.7 (4)
N1—C8—C9—C10	109.5 (3)	C16—C17—C22—C9	0.3 (4)
N1—C8—C9—C22	−69.6 (3)	C16—C17—C22—C21	−179.5 (2)
C8—C9—C10—C11	2.5 (4)	C18—C17—C22—C9	−179.7 (2)
C8—C9—C10—C15	−177.9 (2)	C18—C17—C22—C21	0.6 (4)
C22—C9—C10—C11	−178.4 (2)	C17—C18—C19—C20	0.5 (4)
C22—C9—C10—C15	1.3 (4)	C18—C19—C20—C21	−0.2 (4)
C8—C9—C22—C17	177.8 (2)	C19—C20—C21—C22	0.1 (4)
C8—C9—C22—C21	−2.5 (4)	C20—C21—C22—C9	179.9 (3)
C10—C9—C22—C17	−1.4 (4)	C20—C21—C22—C17	−0.4 (4)
C10—C9—C22—C21	178.3 (2)		

### Hydrogen-bond geometry (Å, º)

*Cg*1, *Cg*2, and *Cg*4 are the centroids of the C2–C7, C9/C10/C15–C17/C22 and C17–C22 rings, respectively. Approximative geometrical parameters are given for the weak N—H..π interaction.

**Table:**

*D*—H···*A*	*D*—H	H···*A*	*D*···*A*	*D*—H···*A*
O1—H1···O2^i^	1.05 (4)	1.58 (3)	2.621 (3)	172 (3)
N1—H1A···*Cg*1^ii^	0.93 (3)	3.49	4.140	129
C4—H4···*Cg*4^iii^	0.93	2.98 (1)	3.752 (3)	141 (1)
C6—H6···*Cg*1^ii^	0.93	2.69 (1)	3.410 (3)	135 (1)
C16—H16···*Cg*4^iv^	0.93	2.83 (1)	3.644 (3)	147 (1)
C18—H18···*Cg*2^iv^	0.93	2.69 (1)	3.452 (3)	140 (1)

Symmetry codes: (i) −*x*+1, −*y*+2, −*z*+2; (ii) −*x*+1, *y*−1/2, −*z*+3/2; (iii) *x*, *y*+1, *z*; (iv) −*x*+2, *y*−1/2, −*z*+3/2.
