# Supplementary material for: Crystal structure and Hirshfeld surface analysis of 4-{[(anthracen-9-yl)meth­yl]amino}­benzoic acid
Source: Acta Crystallogr E Crystallogr Commun. 2020 Jan 1;76(Pt 1):62–5. doi: 10.1107/S2056989019016207 (PMC6944086; doi:10.1107/S2056989019016207)
Supplement: Supplementary file 4 [file e-76-00062-sup3.pdf]

Search Overview

Search:	search4
Date/Time done:	Sat Nov 30 16:10:00 2019
Database(s):	CSD version 5.40 updates (Feb 2019)<br>CSD version 5.40 (November 2018)<br>CSD version 5.40 updates (May 2019)<br>CSD version 5.40 updates (Aug 2019)
Restriction Info:	No refcode restrictions applied
Filters:	None
Percentage Completed:	100%
Number of Hits:	6

Single query used. Search found structures that:

match

Query 1

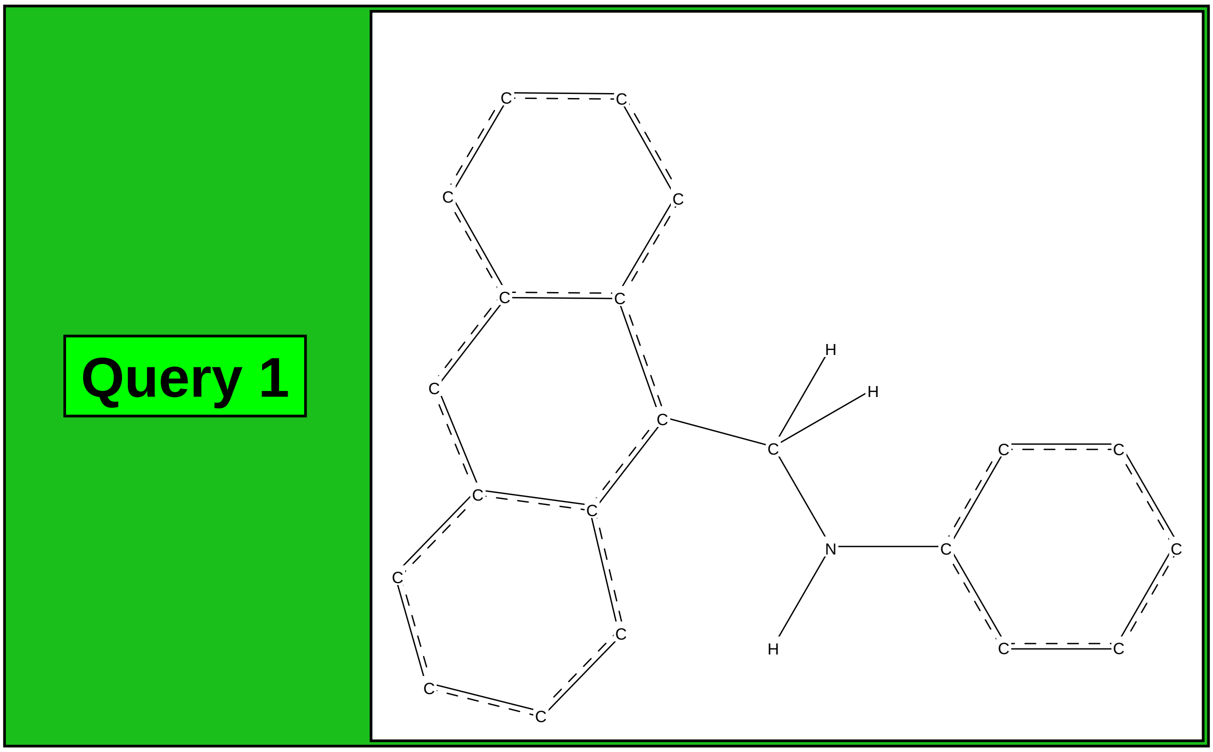

Search: search4 (Sat Nov 30 16:10:00 2019): Hits 1-4

EYUMOC

Reference: Yong Yan, Jun Chen, Ning-Ning Zhang, Ming-Sheng Wang, Cai Sun, Xiu-Shuang Xing, Rong Li, Jian-Gang Xu, Fa-Kun Zheng, Guo-Cong Guo (2016) *Dalton Trans.* ,**45,**18074

Formula: $(C_{26}\ H_{26}\ Cd_1\ N_2\ O_7)n,H_2\ O_1$

Compound Name: catena-((μ-5-[(anthracen-9-ylmethyl)amino]isophthalato)-diaqua-dimethylformamide-cadmium(ii) monohydrate)

Space Group:	P21/c	***Cell:***	***a*** 19.019(4)	***b*** 7.286(1)	***c*** 20.303(4)		
Space Group No.:	14	***(Å,°)***	α 90.00	β 115.34(0)	γ 90.00		
R-Factor (%):	3.36	***Temperature(K):***	293	***Density(g/cm³):***	1.591		

Parameters

Fragment 1
ANG1 (A) 64.305
DIST1 (D) 1.454

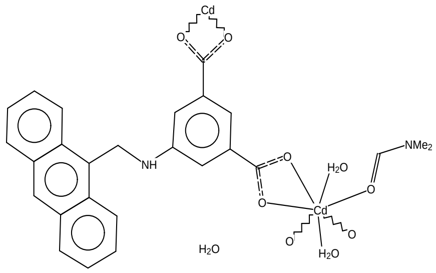

FANHEI

Reference: Jian-Hua Bu, Qi-Yu Zheng, Chuan-Feng Chen, Zhi-Tang Huang (2004) *Org.Lett.* ,**6,**3301

Formula: $C_{55}\ H_{57}\ N_1\ O_7$

Compound Name: 5-(9'-Anthracenylmethylamino)-26,28-diethoxy-25,27-(3,6,9-trioxaundecane-1,11-diolato)calix(4)arene

Space Group:	P21/c	***Cell:***	***a*** 13.815(4)	***b*** 30.518(9)	***c*** 11.346(4)		
Space Group No.:	14	***(Å,°)***	α 90.00	β 109.46(0)	γ 90.00		
R-Factor (%):	7.94	***Temperature(K):***	293	***Density(g/cm³):***	1.243		

Parameters

Fragment 1
ANG1 (A) 84.342
DIST1 (D) 1.451

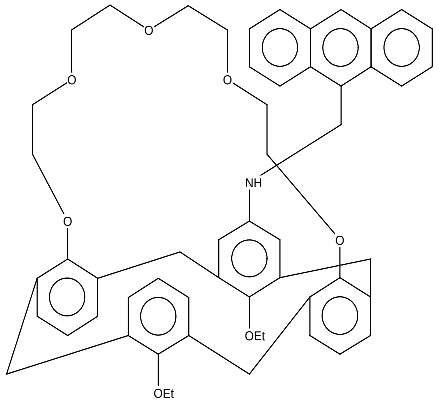

VOLSOG

Reference: Ruchi Singh, J.Mrozinski, P.K.Bharadwaj (2014) *Cryst.Growth Des.* ,**14,**3623

Formula: $(C_{75}\ H_{63}\ Gd_2\ N_5\ O_{16})n,2n(C_3\ H_7\ N_1\ O_1),5.5n(H_2\ O_1)$

Compound Name: catena-(bis(μ3-5-(9-Anthrylmethylamino)isophthalato)-(μ2-5-(9-anthrylmethylamino)isophthalato)-diaqua-bis(dimethylformamide)-di-gadolinium dimethylformamide solvate hydrate)

Space Group:	P-1	***Cell:***	***a*** 16.346(5)	***b*** 17.081(5)	***c*** 17.163(5)		
Space Group No.:	2	***(Å,°)***	α 68.75(0)	β 86.94(0)	γ 66.55(0)		
R-Factor (%):	5.40	***Temperature(K):***	100	***Density(g/cm³):***	1.508		

Parameters

Fragment 1
ANG1 (A) 85.005
DIST1 (D) 1.389

Fragment 2
ANG1 (A) 67.183
DIST1 (D) 1.459

Fragment 3
ANG1 (A) 86.466
DIST1 (D) 1.435

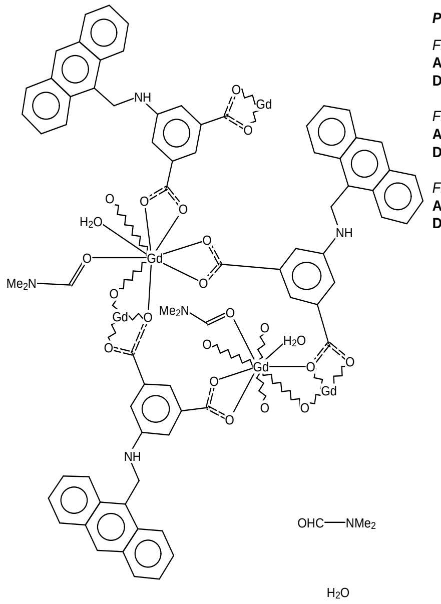

VOLSUM

Reference: Ruchi Singh, J.Mrozinski, P.K.Bharadwaj (2014) *Cryst.Growth Des.* ,**14,**3623

Formula: $(C_{69}\ H_{53}\ Gd_2\ N_3\ O_{16})n,4n(C_3\ H_7\ N_1\ O_1),1.5n(H_2\ O_1)$

Compound Name: catena-((μ4-5-(9-Anthrylmethylamino)isophthalato)-(μ3-5-(9-anthrylmethylamino)isophthalato-O,O',O'',O''')-(μ3-5-(9-anthrylmethylamino)isophthalato-O,O,O',O'',O''')-tetra-aqua-di-gadolinium dimethylformamide solvate sesquihydrate)

Space Group:	P21/c	***Cell:***	***a*** 17.779(5)	***b*** 19.772(5)	***c*** 20.507(5)		
Space Group No.:	14	***(Å,°)***	α 90.00	β 98.49(0)	γ 90.00		
R-Factor (%):	5.39	***Temperature(K):***	100	***Density(g/cm³):***	1.690		

Parameters

Fragment 1
ANG1 (A) 83.995
DIST1 (D) 1.462

Fragment 2
ANG1 (A) 86.863
DIST1 (D) 1.456

Fragment 3
ANG1 (A) 84.918
DIST1 (D) 1.482

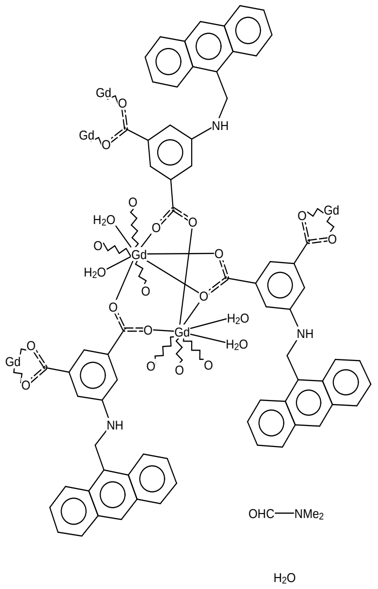

Search: search4 (Sat Nov 30 16:10:00 2019): Hits 5-6

VOLTAT

Reference: Ruchi Singh, J.Mrozinski, P.K.Bharadwaj (2014)
Cryst.Growth Des. ,**14,**3623

Formula: $(C_{75} H_{61} Gd_2 N_5 O_{15})n,2n(C_3 H_7 N_1 O_1),2n(C_1 H_2 Cl_2),5n(H_2 O_1)$

Compound Name: catena-(tris(μ_3-5-(9-Anthrylmethylamino)isophthalato)-aqua-bis(dimethylformamide)-di-gadolinium dichloromethane dimethylformamide solvate pentahydrate)

Space Group:	P-1	***Cell:***	***a*** 15.418(5)	***b*** 16.274(5)	***c*** 17.876(5)
Space Group No.:	2	***(Å,°)***	α 109.84(0)	β 93.62(0)	γ 111.14(0)

R-Factor (%): 7.00 ***Temperature(K):*** 100 ***Density(g/cm^3):*** 1.721

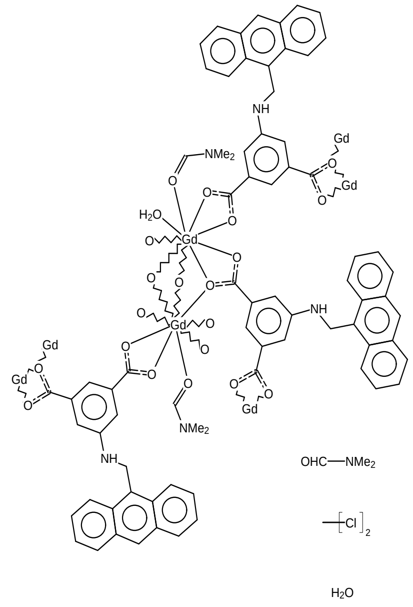

Parameters

Fragment 1
ANG1 (A) 67.514
DIST1 (D) 1.494

Fragment 2
ANG1 (A) 82.137
DIST1 (D) 1.472

Fragment 3
ANG1 (A) 86.494
DIST1 (D) 1.455

VOLTIB

Reference: Ruchi Singh, J.Mrozinski, P.K.Bharadwaj (2014)
Cryst.Growth Des. ,**14,**3623

Formula: $(C_{51} H_{41} Gd_1 N_3 O_9)n$

Compound Name: catena-(bis(μ_3-5-(9-Anthrylmethylamino)isophthalato)-diethylformamide-gadolinium)

Space Group:	P21/c	***Cell:***	***a*** 25.571(5)	***b*** 10.862(5)	***c*** 16.025(5)
Space Group No.:	14	***(Å,°)***	α 90.00	β 105.82(0)	γ 90.00

R-Factor (%): 4.22 ***Temperature(K):*** 100 ***Density(g/cm^3):*** 1.547

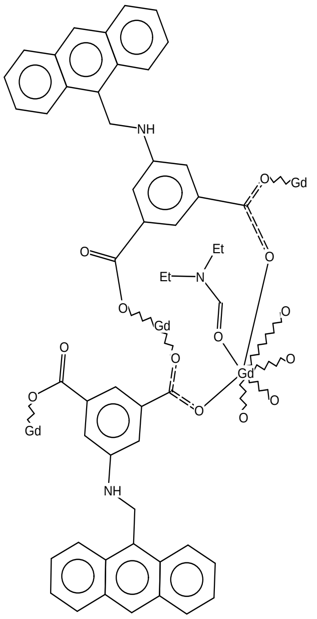

Parameters

Fragment 1
ANG1 (A) 65.804
DIST1 (D) 1.457

Fragment 2
ANG1 (A) 54.676
DIST1 (D) 1.461
