# Supplementary material for: Crystal structure and Hirshfeld surface analysis of 4-{[(anthracen-9-yl)meth­yl]amino}­benzoic acid
Source: Acta Crystallogr E Crystallogr Commun. 2020 Jan 1;76(Pt 1):62–5. doi: 10.1107/S2056989019016207 (PMC6944086; doi:10.1107/S2056989019016207)

Search Overview

Search:	search7
Date/Time done:	Sat Nov 30 16:11:53 2019
Database(s):	CSD version 5.40 updates (Feb 2019)<br>CSD version 5.40 (November 2018)<br>CSD version 5.40 updates (May 2019)<br>CSD version 5.40 updates (Aug 2019)
Restriction Info:	No refcode restrictions applied
Filters:	None
Percentage Completed:	100%
Number of Hits:	21

Single query used. Search found structures that:

match

Query 1

Query 1

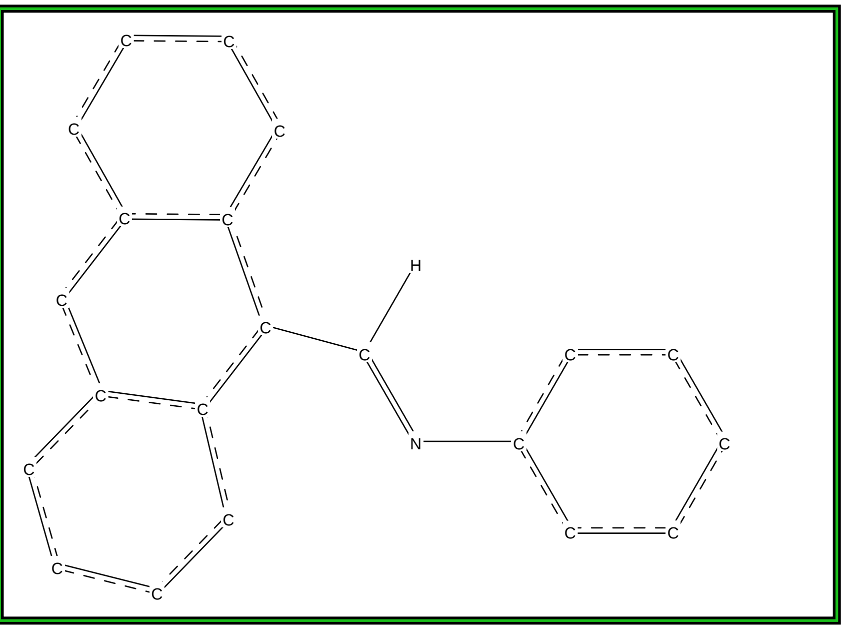

Search: search7 (Sat Nov 30 16:11:53 2019): Hits 1-4

AXODAS

Reference: Wai-Yeung Wong, Guo-Liang Lu, Li Liu, Jian-Xin Shi, Zhenyang Lin (2004) *Eur.J.Inorg.Chem.* ,2066

Formula: $C_{22}\,H_{17}\,N_1\,O_1$

Compound Name: N-(9-Anthrylmethylene)-1-amino-4-methoxybenzene

Space Group:	P-1	***Cell:***	***a***	9.397(0)	***b***	10.679(0)	***c***	17.090(1)
Space Group No.:	2	***(Å,°)***	α	88.63(0)	β	75.53(0)	γ	85.59(0)

R-Factor (%): 4.46 ***Temperature(K):*** 293 ***Density(g/cm^3):*** 1.249

Parameters

Fragment 1
ANG1 (A) 68.023
DIST1 (D) 1.252

Fragment 2
ANG1 (A) 66.319
DIST1 (D) 1.212

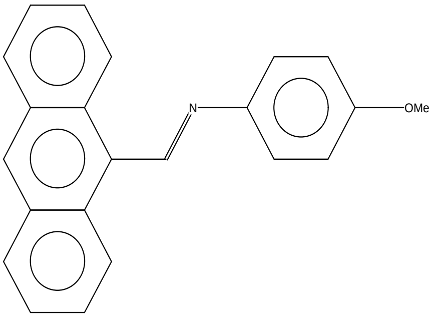

AXODUM

Reference: Wai-Yeung Wong, Guo-Liang Lu, Li Liu, Jian-Xin Shi, Zhenyang Lin (2004) *Eur.J.Inorg.Chem.* ,2066

Formula: $C_{30}\,H_{21}\,Hg_1\,N_1\,O_1$

Compound Name: (N-(9-Anthrylmethylene)-2-amino-5-methoxyphenyl)-(2-phenylethynyl)-mercury

Space Group:	P65	***Cell:***	***a***	26.103(2)	***b***	26.103(2)	***c***	5.942(0)
Space Group No.:	170	***(Å,°)***	α	90.00	β	90.00	γ	120.00

R-Factor (%): 5.10 ***Temperature(K):*** 293 ***Density(g/cm^3):*** 1.739

Parameters

Fragment 1
ANG1 (A) 11.204
DIST1 (D) 1.257

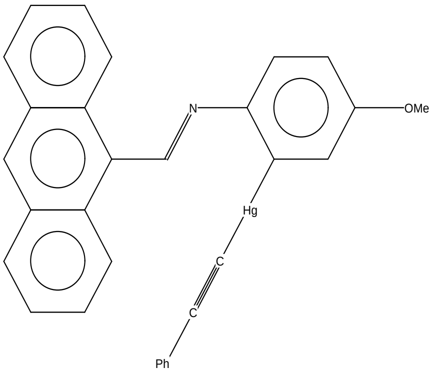

AXOFAU

Reference: Wai-Yeung Wong, Guo-Liang Lu, Li Liu, Jian-Xin Shi, Zhenyang Lin (2004) *Eur.J.Inorg.Chem.* ,2066

Formula: $C_{34}\,H_{25}\,Fe_1\,Hg_1\,N_1\,O_1$

Compound Name: (N-(9-Anthrylmethylene)-2-amino-5-methoxyphenyl)-(2-ferrocenylethynyl)-mercury

Space Group:	P21/c	***Cell:***	***a***	15.296(0)	***b***	12.760(0)	***c***	13.580(0)
Space Group No.:	14	***(Å,°)***	α	90.00	β	95.66(0)	γ	90.00

R-Factor (%): 2.84 ***Temperature(K):*** 293 ***Density(g/cm^3):*** 1.813

Parameters

Fragment 1
ANG1 (A) 3.869
DIST1 (D) 1.196

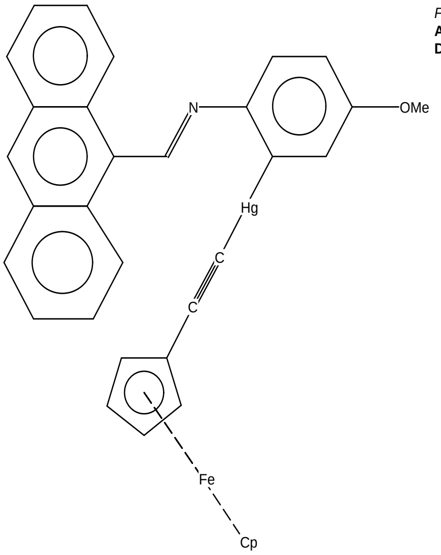

AZORUD

Reference: K.Geetha, D.K.A.P.Kumar, D.Lakshmanan, R.Savitha, S.Murugavel (2011) *Acta Crystallogr.,Sect.E:Struct.Rep.Online* ,**67,** o2577

Formula: $C_{21}\,H_{14}\,N_2\,O_2$

Compound Name: (E)-N-(9-Anthrylmethylene)-4-nitroaniline

Space Group:	P-1	***Cell:***	***a***	8.363(0)	***b***	8.905(0)	***c***	11.512(0)
Space Group No.:	2	***(Å,°)***	α	75.23(0)	β	84.54(0)	γ	75.05(0)

R-Factor (%): 6.99 ***Temperature(K):*** 293 ***Density(g/cm^3):*** 1.354

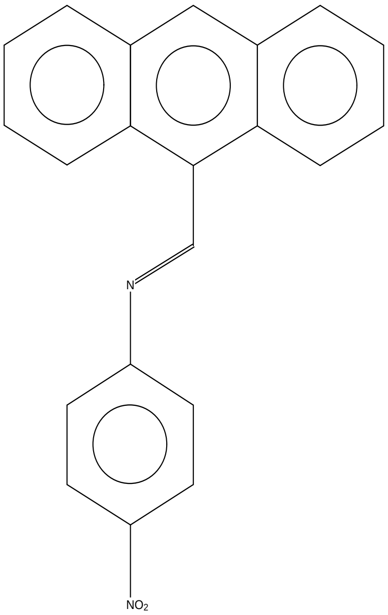

Parameters

Fragment 1
ANG1 (A) 73.564
DIST1 (D) 1.245

Search: search7 (Sat Nov 30 16:11:53 2019): Hits 5-8

BUHWAD

Reference: V.Chandrasekhar, A.Kumar, M.D.Pandey (2010) *J.Organomet.Chem.* ,**695,**74

Formula: $C_{29}\ H_{23}\ Cl_2\ N_1\ O_1\ Te_1$

Compound Name: N-(9-Anthrylmethylene)-2-(dichloro(4-methoxyphenyl)-λ^4-tellanyl)-4-methylaniline

Space Group:	P-1	***Cell:***	***a*** 8.990(5)	***b*** 10.213(5)	***c*** 13.234(5)		
Space Group No.:	2	***(Å,°)***	α 91.64(0)	β 93.22(0)	γ 95.52(0)		
R-Factor (%):	4.98	***Temperature(K):***	100	***Density(g/cm^3):***	1.651		

Parameters

Fragment 1
ANG1 (A) 89.291
DIST1 (D) 1.287

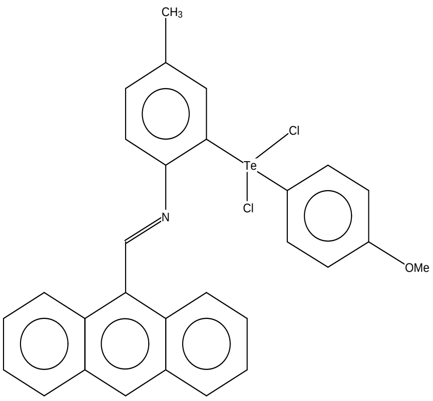

BUHWEH

Reference: V.Chandrasekhar, A.Kumar, M.D.Pandey (2010) *J.Organomet.Chem.* ,**695,**74

Formula: $C_{32}\ H_{23}\ Cl_2\ N_1\ Te_1, C_1\ O_2, 2.5(H_2\ O_1)$

Compound Name: N-(9-Anthrylmethylene)-2-(dichloro(1-naphthyl)-λ^4-tellanyl)-4-methylaniline carbon dioxide hydrate

Space Group:	P-1	***Cell:***	***a*** 7.618(3)	***b*** 13.128(4)	***c*** 15.219(5)		
Space Group No.:	2	***(Å,°)***	α 105.68(0)	β 104.38(0)	γ 95.87(0)		
R-Factor (%):	6.54	***Temperature(K):***	100	***Density(g/cm^3):***	1.687		

Parameters

Fragment 1
ANG1 (A) 18.075
DIST1 (D) 1.273

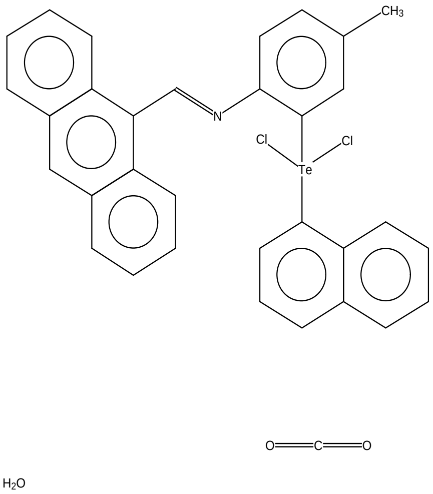

GISTOS

Reference: D.Gayathri, D.Velmurugan, K.Ravikumar, S.Devaraj, M.Kandaswamy (2008) *Acta Crystallogr.,Sect.E:Struct.Rep.Online* ,**64,** o408

Formula: $C_{28}\ H_{21}\ N_3\ S_1$

Compound Name: 1-(2-((Anthracen-10-yl)methyleneamino)phenyl)-3-phenylthiourea

Space Group:	P-1	***Cell:***	***a*** 6.615(0)	***b*** 14.315(1)	***c*** 23.823(2)		
Space Group No.:	2	***(Å,°)***	α 74.05(0)	β 88.70(0)	γ 87.42(0)		
R-Factor (%):	4.67	***Temperature(K):***	293	***Density(g/cm^3):***	1.323		

Parameters

Fragment 1
ANG1 (A) 80.612
DIST1 (D) 1.274

Fragment 2
ANG1 (A) 82.777
DIST1 (D) 1.259

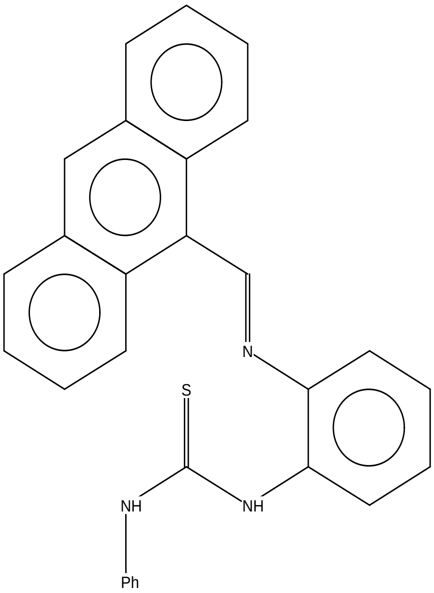

LOBROK

Reference: Jin Zhou, Qi-Bao Wang, Hongjian Sun (2008) *Acta Crystallogr.,Sect.E:Struct.Rep.Online* ,**64,**m688

Formula: $C_{54}\ H_{52}\ Cl_2\ N_2\ Pd_2$

Compound Name: bis(μ_2-chloro)-bis((9-(2,6-di-isopropylphenyliminomethyl)-anthracen-1-yl)-palladium(ii))

Space Group:	P21/n	***Cell:***	***a*** 12.300(0)	***b*** 12.984(0)	***c*** 15.456(0)		
Space Group No.:	14	***(Å,°)***	α 90.00	β 110.36(0)	γ 90.00		
R-Factor (%):	4.97	***Temperature(K):***	298	***Density(g/cm^3):***	1.453		

Parameters

Fragment 1
ANG1 (A) 69.465
DIST1 (D) 1.293

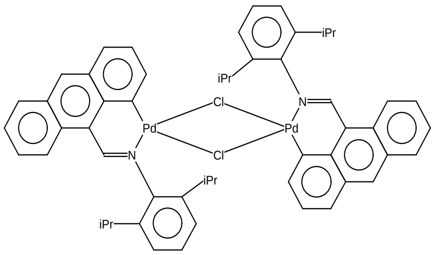

Search: search7 (Sat Nov 30 16:11:53 2019): Hits 9-12

OCOLUQ

Reference: M.S.H.Faizi, A.Haque, M.Ahmad, I.A.Golenya (2017) *Acta Crystallogr.,Sect.E:Cryst.Commun.* ,**73,**137

Formula: $C_{27} H_{20} N_2$

Compound Name: 4-((9-anthrylmethylene)amino)-N-phenylaniline

Space Group: P21/c ***Cell:*** ***a*** 11.155(0) ***b*** 45.224(3) ***c*** 11.586(0)
Space Group No.: 14 ***(Å,°)*** α 90.00 β 96.64(0) γ 90.00

R-Factor (%): 5.21 ***Temperature(K):*** 100 ***Density(g/cm^3):*** 1.278

Parameters

Fragment 1
ANG1 (A) 80.101
DIST1 (D) 1.276

Fragment 2
ANG1 (A) 82.676
DIST1 (D) 1.278

Fragment 3
ANG1 (A) 85.011
DIST1 (D) 1.271

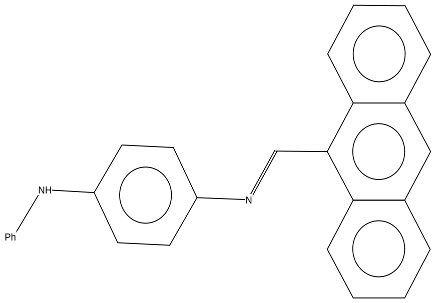

OHISUV

Reference: L.Marin, A.van der Lee, S.Shova, A.Arvinte, M.Barboiu (2015) *New J.Chem.* ,**39,**6404

Formula: $C_{29} H_{31} N_1,0.08(C_2 H_3 N_1)$

Compound Name: 1-(9-Anthryl)-N-(4-octylphenyl)methanimine acetonitrile solvate

Space Group: R-3 ***Cell:*** ***a*** 46.312(3) ***b*** 46.312(3) ***c*** 5.627(0)
Space Group No.: 148 ***(Å,°)*** α 90.00 β 90.00 γ 120.00

R-Factor (%): 4.24 ***Temperature(K):*** 175 ***Density(g/cm^3):*** 1.135

Parameters

Fragment 1
ANG1 (A) 7.057
DIST1 (D) 1.277

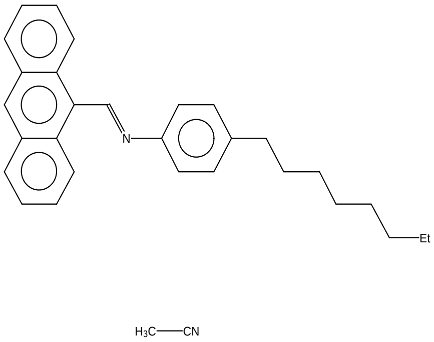

OHITAC

Reference: L.Marin, A.van der Lee, S.Shova, A.Arvinte, M.Barboiu (2015) *New J.Chem.* ,**39,**6404

Formula: $C_{25} H_{23} N_1$

Compound Name: 1-(9-Anthryl)-N-(4-butylphenyl)methanimine

Space Group: P-1 ***Cell:*** ***a*** 8.215(0) ***b*** 9.577(0) ***c*** 12.801(0)
Space Group No.: 2 ***(Å,°)*** α 73.64(0) β 73.32(0) γ 89.65(0)

R-Factor (%): 6.30 ***Temperature(K):*** 175 ***Density(g/cm^3):*** 1.215

Parameters

Fragment 1
ANG1 (A) 78.097
DIST1 (D) 1.278

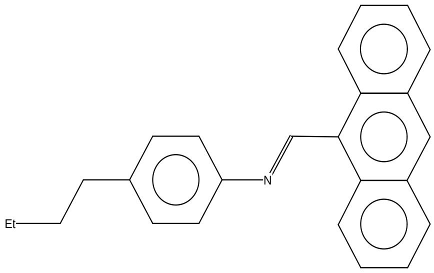

OHITEG

Reference: L.Marin, A.van der Lee, S.Shova, A.Arvinte, M.Barboiu (2015) *New J.Chem.* ,**39,**6404

Formula: $C_{29} H_{29} N_1 O_5$

Compound Name: 1-(9-Anthryl)-N-(2,3,5,6,8,9,11,12-octahydro-1,4,7,10,13-benzopentaoxacyclopentadecin-15-yl)methanimine

Space Group: P21/c ***Cell:*** ***a*** 19.839(0) ***b*** 8.767(0) ***c*** 13.870(0)
Space Group No.: 14 ***(Å,°)*** α 90.00 β 101.79(0) γ 90.00

R-Factor (%): 6.00 ***Temperature(K):*** 175 ***Density(g/cm^3):*** 1.326

Parameters

Fragment 1
ANG1 (A) 87.552
DIST1 (D) 1.273

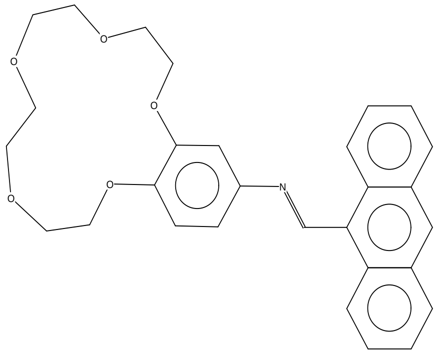

Search: search7 (Sat Nov 30 16:11:53 2019): Hits 13-16

OWAFEY

Reference: A.Villapando, F.R.Fronczek, R.Isovitsch (2011)
J.Chem.Cryst. ,**41,**1342

Formula: $C_{22}\ H_{17}\ N_1\ O_1$

Compound Name: 2-((9-Anthrylmethylene)amino)-5-methylphenol

Space Group:	P-1	***Cell:***	***a*** 8.553(0)	***b*** 14.093(1)	***c*** 14.938(1)
Space Group No.:	2	***(Å,°)***	α 104.20(0)	β 106.48(0)	γ 105.09(0)
R-Factor (%):	3.30	***Temperature(K):*** 90	***Density(g/cm³):*** 1.322		

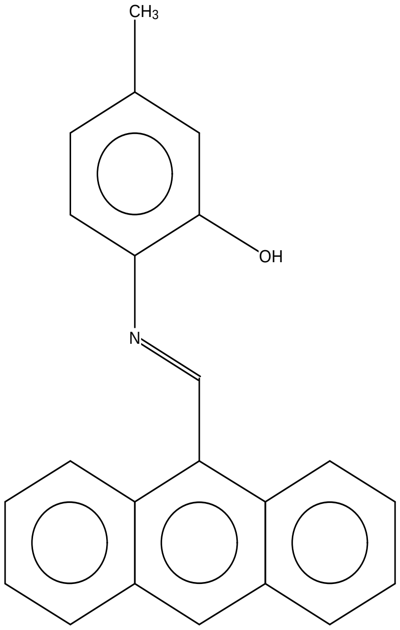

Parameters

Fragment 1
ANG1 (A) 67.325
DIST1 (D) 1.279

Fragment 2
ANG1 (A) 78.076
DIST1 (D) 1.279

TUPGIV

Reference: A.Villalpando, F.R.Fronczek, R.Isovitsch (2010)
Acta Crystallogr.,Sect.E:Struct.Rep.Online ,**66,**o1353

Formula: $C_{22}\ H_{17}\ N_1\ O_1$

Compound Name: 2-(9-Anthrylmethylideneamino)-4-methylphenol

Space Group:	P-1	***Cell:***	***a*** 8.604(1)	***b*** 12.839(3)	***c*** 15.015(3)
Space Group No.:	2	***(Å,°)***	α 94.51(0)	β 97.16(1)	γ 106.49(1)
R-Factor (%):	5.40	***Temperature(K):*** 90	***Density(g/cm³):*** 1.320		

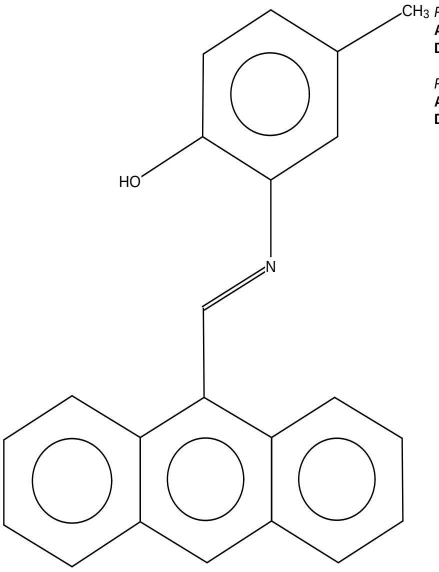

Parameters

Fragment 1
ANG1 (A) 72.701
DIST1 (D) 1.279

Fragment 2
ANG1 (A) 86.049
DIST1 (D) 1.280

UFOZOE

Reference: C.M.Vogels, L.G.Nikolcheva, D.W.Norman, H.A.Spinney, A.Decken, M.O.Baerlocher, F.J.Baerlocher, S.A.Westcott (2001)
Can.J.Chem. ,**79,**1115

Formula: $C_{27}\ H_{26}\ B_1\ N_1\ O_2$

Compound Name: 2-(3-(9-Anthracenylmethyleneamino)phenyl)-4,4,5,5-tetramethyl-1,3,2-dioxaborolan

Space Group:	P-1	***Cell:***	***a*** 9.679(0)	***b*** 10.740(0)	***c*** 11.535(0)
Space Group No.:	2	***(Å,°)***	α 105.19(0)	β 97.30(0)	γ 102.15(0)
R-Factor (%):	4.76	***Temperature(K):*** 173	***Density(g/cm³):*** 1.219		

Parameters

Fragment 1
ANG1 (A) 87.350
DIST1 (D) 1.276

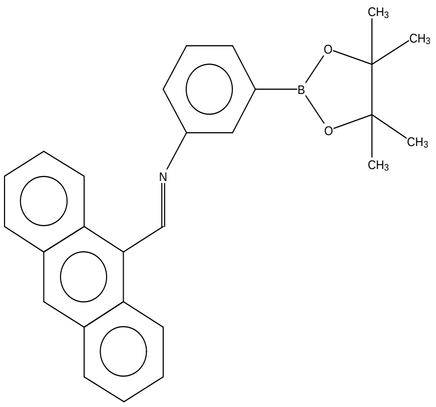

VIMBOK

Reference: J.P.Ward, J.M.White, C.G.Young (2013) *Tetrahedron* ,**69,** 8824

Formula: $C_{74}\ H_{74}\ N_2\ O_4,3(C_1\ H_2\ Cl_2)$

Compound Name: 11,23-bis((9-anthrylmethylene)amino)-26,28-dibutoxy-5,17-di-t-butylpentacyclo[19.3.1.1^{3,7}.1^{9,13}.1^{15,19}]octacosa-1(25),3(28),4,6,9(27),10,12,15(26),16,18,21,23-dodecaene-25,27-diol dichloromethane solvate

Synonym: 5,17-bis((4-anthracen-9-yl)methyleneamino)-11,23-di-t-butyl-25,27-di-n-butoxy-26,28-dihydroxycalix[4]arene dichloromethane solvate

Space Group:	P-1	***Cell:***	***a*** 13.357(1)	***b*** 17.201(0)	***c*** 18.045(1)
Space Group No.:	2	***(Å,°)***	α 114.62(0)	β 108.78(0)	γ 92.06(0)
R-Factor (%):	8.27	***Temperature(K):*** 130	***Density(g/cm³):*** 1.244		

Parameters

Fragment 1
ANG1 (A) 85.177
DIST1 (D) 1.264

Fragment 2
ANG1 (A) 70.976
DIST1 (D) 1.289

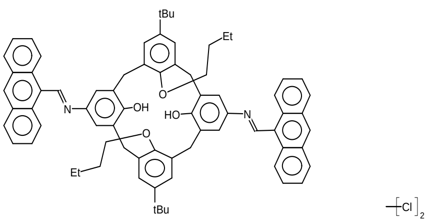

Search: search7 (Sat Nov 30 16:11:53 2019): Hits 17-20

ZEKLAE

Reference: S.Guha, S.Lohar, A.Banerjee, A.Sahana, S.K.Mukhopadhyay, J.S.Matalobos, D.Das (2012) *Analytical Methods* , **4,**3163

Formula: $C_{24} H_{15} N_1 O_2$

Compound Name: 6-((9-Anthrylmethylene)amino)-2H-chromen-2-one

Space Group:	P21/n	***Cell:***	***a***	6.661	***b***	30.083	***c***	17.635
Space Group No.:	14	***(Å,°)***	α	90.00	β	99.52	γ	90.00

R-Factor (%): 6.28 ***Temperature(K):*** 293 ***Density(g/cm^3):*** 1.332

Parameters

Fragment 1
ANG1 (A) 67.111
DIST1 (D) 1.282

Fragment 2
ANG1 (A) 73.154
DIST1 (D) 1.280

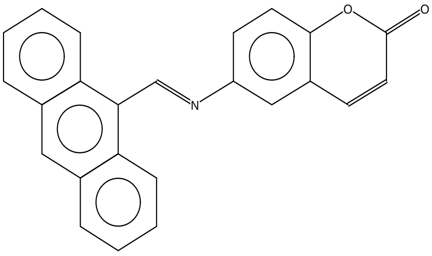

TITNES

Reference: G.P.Junor, E.A.Romero, Xi Chen, R.Jazzar, G.Bertrand (2019) *Angew.Chem.,Int.Ed.* ,**58,**2875

Formula: $C_{21} H_{15} N_1$

Compound Name: 1-(anthracen-9-yl)-N-phenylmethanimine

Space Group:	P21/n	***Cell:***	***a***	4.984(0)	***b***	11.529(0)	***c***	24.972(0)
Space Group No.:	14	***(Å,°)***	α	90.00	β	92.00(0)	γ	90.00

R-Factor (%): 3.08 ***Temperature(K):*** 100 ***Density(g/cm^3):*** 1.303

Parameters

Fragment 1
ANG1 (A) 88.474
DIST1 (D) 1.279

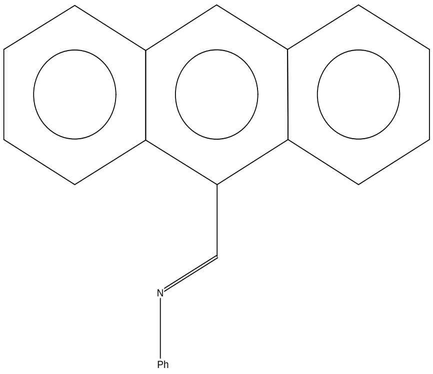

YIVQAY

Reference: M.Barwiolek, A.Wojtczak, A.Kozakiewicz, M.Babinska, A.Tafelska-Kaczmarek, E.Larsen, E.Szlyk (2019) *J.Lumin.* ,**211,**88

Formula: $C_{21} H_{16} N_2$

Compound Name: 2-{[(anthracen-9-yl)methylidene]amino}aniline

Space Group:	P21/c	***Cell:***	***a***	10.567(1)	***b***	13.101(1)	***c***	11.506(1)
Space Group No.:	14	***(Å,°)***	α	90.00	β	104.85(1)	γ	90.00

R-Factor (%): 4.25 ***Temperature(K):*** 293 ***Density(g/cm^3):*** 1.278

Parameters

Fragment 1
ANG1 (A) 72.127
DIST1 (D) 1.265

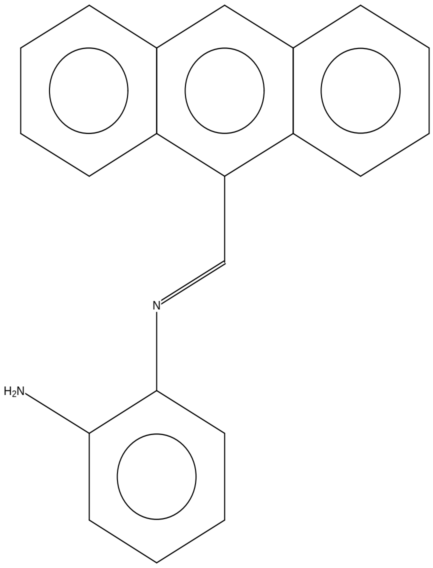

WOKGEL

Reference: R.Kumar, H.Aggarwal, R.Bhowal, D.Chopra, A.Srivastava (2019) *Chem.-Eur.J.* ,**25,**10756

Formula: $C_{49} H_{33} N_5 O_2$

Compound Name: N^2,N^6-bis(2-{[(anthracen-9-yl)methylidene]amino}phenyl)pyridine-2,6-dicarboxamide

Space Group:	Fdd2	***Cell:***	***a***	12.427(1)	***b***	20.670(1)	***c***	28.094(4)
Space Group No.:	43	***(Å,°)***	α	90.00	β	90.00	γ	90.00

R-Factor (%): 8.25 ***Temperature(K):*** 146 ***Density(g/cm^3):*** 1.332

Parameters

Fragment 1
ANG1 (A) 46.106
DIST1 (D) 1.278

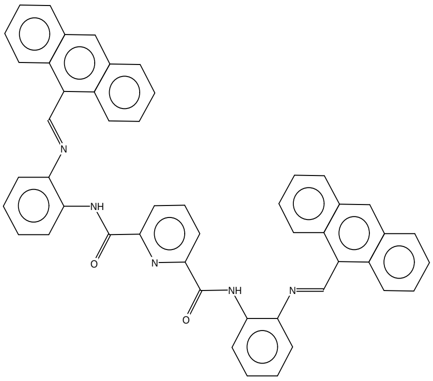

Search: search7 (Sat Nov 30 16:11:53 2019): Hit 21

WOMTOK

Reference:	R.Kumar, H.Aggarwal, R.Bhowal, D.Chopra, A.Srivastava<br>(2019) *Chem.-Eur.J.* ,**25,**10756
Formula:	C_{49} H_{33} N_5 O_2,C_{10} H_2 N_4
Compound Name:	N^2,N^6-bis(2-{[(anthracen-9-yl)methylidene]amino}phenyl)pyridine-2,6-dicarboxamide benzene-1,2,4,5-tetracarbonitrile
Synonym:	Heli(aza)cene 1,2,4,5-tetracyanobenzene

Space Group:	C2/c	***Cell:***	***a***	19.693(0)	***b***	16.745(0)	***c***	13.713(0)
Space Group No.:	15	***(Å,°)***	α	90.00	β	99.19(0)	γ	90.00

R-Factor (%):	12.12	***Temperature(K):***	120	***Density(g/cm³):***	1.342

Parameters

Fragment 1

**ANG1 (A)**	12.807
**DIST1 (D)**	1.185

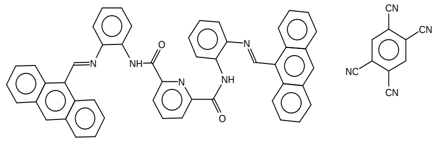

Supplement: Supplementary file 5 [file e-76-00062-sup4.pdf]
